# Mimicking of Phase I Metabolism Reactions of Molindone by HLM and Photocatalytic Methods with the Use of UHPLC-MS/MS

**DOI:** 10.3390/molecules25061367

**Published:** 2020-03-17

**Authors:** Maciej Gawlik, Vladimir Savic, Milos Jovanovic, Robert Skibiński

**Affiliations:** 1 Department of Medicinal Chemistry, Faculty of Pharmacy, Medical University of Lublin, Jaczewskiego 4, 20-090 Lublin, Poland; maciej.gawlik@umlub.pl; 2 Department of Organic Chemistry, Faculty of Pharmacy, University of Belgrade, Vojvode Stepe 450, 11221 Belgrade, Serbia; vladimir.savic@pharmacy.bg.ac.rs (V.S.); milos.jovanovic@pharmacy.bg.ac.rs (M.J.)

**Keywords:** photocatalysis, metabolites, HLM, mass spectrometry, chemometric analysis

## Abstract

Establishing the metabolism pathway of the drug undergoing the hepatic biotransformation pathway is one of the most important aspects in the preclinical discovery process since the presence of toxic or reactive metabolites may result in drug withdrawal from the market. In this study, we present the structural elucidation of six, not described yet, metabolites of an antipsychotic molecule: molindone. The elucidation of metabolites was supported with a novel photocatalytical approach with the use of WO_3_ and WS_2_ assisted photochemical reactions. An UHPLC-ESI-Q-TOF combined system was used for the registration of all obtained metabolite profiles as well as to record the high resolution fragmentation spectra of the observed transformation products. As a reference in the in vitro metabolism simulation method, the incubation with human liver microsomes was used. Chemometric comparison of the obtained profiles pointed out the use of the WO_3_ approach as being more convenient in the field of drug metabolism studies. Moreover, the photocatalysis was used in the direction of the main drug metabolite synthesis in order to further isolation and characterization.

## 1. Introduction

Molindone (3-ethyl-2-methyl-5-(morpholin-4-ylmethyl)-1,5,6,7-tetrahydroindol-4-one) is a medicine originally and mainly used in schizophrenia treatment. In addition, its use is also considered in ADHD therapy (attention-deficit/hyperactivity disorder) [1,2,3]. The mechanism of molindone action assumes D_2S_, D_2L_, D_5_ dopamine, and 5-HT_2B_ serotonin receptor antagonist activity and remains strictly in correlation with the dopamine hypothesis of schizophrenia [4,5,6,7,8,9]. It should also be noted that patients treated with molindone are exposed to the occurrence of side effects, for instance, tardive dyskinesia or akathisia, according to the commonly known mechanism of neuroleptics dopamine antagonistic potential [10,11,12]. The drug is considered to undergo the hepatic metabolism pathway and its use in treatment should be monitored due to possible hepatotoxic impact [13,14]. Nevertheless, molindone reveals activity corresponding to leading antipsychotics and does not influence weight gain, which is an infrequent property in the field of neuroleptic drugs [15,16]. As molindone has been introduced anew to market and is used in schizophrenia therapy, the issue of the hepatotoxicity origin is still not better known.

Moreover, the liver is an organ that is mainly responsible for drug metabolism, intoxification, and preparing to excrete intermediates from the human organism. The medicine molecule participating in biochemical processes changes its physicochemical properties, which can be responsible for the formation of toxic metabolites [17,18,19,20]. As these multidirectional processes occur and at the same time lead to the formation of many derivatives, a detailed understanding of the transformation pathways of a medicinal compound as well as metabolites brings knowledge of both the mechanisms of drug action and underlies further research on the drug. The activity that the liver shows in the metabolism of medicines has become the basis for developing an in vitro assay for determining drug metabolism [21]. The human liver microsomes (HLMs) used for this purpose provide satisfactory results with reference to drug molecules undergoing the hepatic pathway [22,23,24]. In spite of its unequivocal usefulness in drug metabolism studies, this approach also reflects minor drawbacks. Due to the co-presence of biological interferences such as cell matrix, proteins, and phospholipids, the use of HLM incubation in metabolite isolation is a complex process and requires a time-consuming workflow [25]. Additionally, the explicit decrease in reaction efficiency relative to the incubation time further limits the use of this method as an simple and effortless source of metabolites [26].

The further attempts to decrease severe costs associated with drug development studies have contributed to involve photo-assisted catalysis with the use of metal oxide nanoparticles in metabolism simulation processes [27,28,29,30]. This new approach benefits from economic efficiency, a simple procedure, and also requires less human resources. The principle of this method is based on metal oxide semiconductor properties with the ability to create reactive oxygen species (ROS) in aqueous solution and under simultaneous adequate UV irradiation [31,32]. Generated radicals act as a strong oxidizing agents and cause molecule oxidation in the presence of the tested drug substance in solution. Despite the fact that heterogeneous photocatalysis was originally applied in drug metabolism studies with the use of model substance Degussa P25 titanium dioxide (TiO_2_), tungsten oxide (WO_3_) assisted photocatalysis turned out to provide satisfactory and promising results [33,34]. The synthesis and isolation of metabolites is a distinct issue. Since the conventional approach of obtaining metabolites assumes organic synthesis, some modern techniques such as biosynthesis, enzymatic methods, and electrochemical (EC) synthetic reactions are particularly gaining attention [35,36,37,38,39,40]. In this context, the photocatalytic transformation of drugs in order to further metabolite isolation seems to become a promising tool. This method simplifies the procedure and given the lack of the use of any organic solvents, conforms to the principles of green chemistry.

The aim of this study was to develop a simple, fast, and cheap method for preliminary drug metabolism research. The detailed molindone metabolism pathway was established in accordance with WO_3_ and tungsten disulfide (WS_2_) ssisted photocatalysis. Furthermore, the feasibility of study, assuming the use of the photocatalytic protocol in order to further metabolite isolation, was successfully confirmed. In addition, structural elucidation of molindone metabolites was carried out based on the ultra-high-pressure liquid chromatography-electrospray ionization-quadrupole time-of-flight-mass spectrometry (UHPLC-ESI-Q-TOF MS) combined system, which is a highly useful tool in drug metabolism studies [41,42,43,44].

## 2. Results and Discussion

### 2.1. Photocatalytic Degradation Kinetics

**Figure 1:**
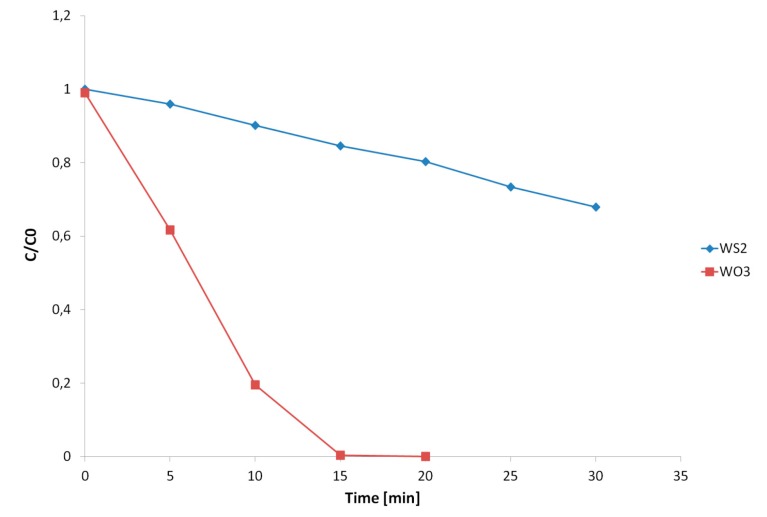
Kinetics of molindone photocatalytic decomposition presented as a normalized concentration (C/C_0_) against time (min).

In the pilot study, the adsorption of molindone on WO_3_ and WS_2_ was tested in the 0–60 min time range and no significant differences were found in the concentrations of the analyzed compound. Therefore, a 60 min adsorption–desorption equilibrium time was defined for both catalyzed samples. Subsequently, the degradation kinetics were studied within the given time ranges of 0–20 min for WO_3_ and 0–30 min for the WS_2_ photocatalytic experiments. The obtained results show that the photocatalytic decomposition of molindone yielded pseudo-first kinetics for the WO_3_ assisted (*k* = 0.1634 min^−1^, t_1/2_ = 4.3 min, r = 0.9532) and WS_2_ assisted (*k* = 0.0129 min^−1^, t_1/2_ = 63 min, r = 0.9948) photochemical reaction (Figure 1).

### 2.2. Multivariate Comparison of Human Liver Microsome (HLM) Metabolites and Photocatalytic Products

**Figure 2:**
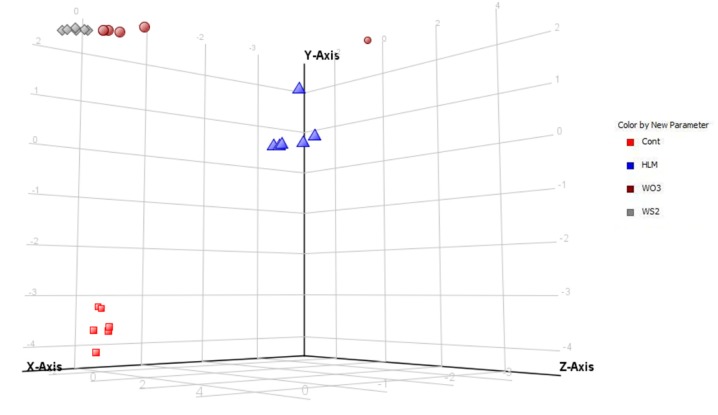
PCA plot of HLM (blue triangles), WS_2_ (grey diamonds), and WO_3_ (brown spheres) with the control group (red squares) profiles of molindone.

In order to perform a preliminary comparison between WS_2_ and WO_3_ photo-assisted catalysis with regard to the HLM metabolite profile of molindone, a multivariate chemometric analysis was performed. All of the obtained chromatographic profiles (24 chromatograms) registered in time-of-flight (MS) mode were aligned with Mass Profiler Professional (MPP) software, giving 176 entities. After a build-in MPP filtration including sample abundance and the Mann–Whitney u-test (*p* < 0.05, FC > 1.1), 17 entities were finally selected for the chemometric study. The PCA analysis based on this data showed a visible categorization of all of the analyzed groups of the registered metabolic profiles (Figure 2). Two types of photocatalytic profiles remained in a short distance from each other, however, the samples belonging to WO_3_ inducted photocatalysis were closer to the HLM samples. Negative control samples (Cont) stood out from the other profiles, which confirmed the occurrence of metabolic reactions. The achieved results suggest that WO_3_ photocatalytic profiles are substantially more similar to hepatic metabolism profiles than WS_2_. In the presented principal component analysis (PCA), the first three components (PC) explained 95.0% of the total variance.

Taking this into account, the proposed WO_3_ photocatalytic method could be considered as a more suitable approach for mimicking the phase I metabolism reactions. Moreover, considering the degradation kinetic parameters (Section 2.1), this catalyst is also more suitable for the production and isolation of the main metabolites of molindone. 

### 2.3. Metabolites Identification

**Figure 3:**
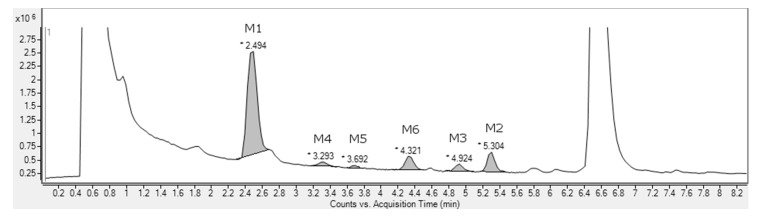
Total ion chromatogram obtained for HLM experiment.

Six metabolites of molindone were identified in this study. Metabolite structures were elucidated by UHPLC-ESI-Q-TOF analysis with the use of recorded high resolution MS/MS spectra. The fragmentation patterns of molindone and its metabolites are summarized in Table 1, and an example of the total ion chromatogram obtained for the HLM experiment is presented in Figure 3.

In order to perform accurate structural elucidation of the main metabolite (M1), an additional WO_3_ assisted photocatalytic experiment was performed. Based on our preliminary photocatalysis optimization process, the loading level of the parent compound as well as the irradiation time were increased, which provided the opportunity to obtain a semi-preparative level of this metabolite (Section 3.2). Next, the ultra-fast liquid chromatography (UFLC) reversed-phase system was used for M1 isolation; after evaporation and dissolution in deuterated methanol, 1H NMR spectroscopy was carried out. 

The protonated molecular ion for molindone (Figure S1) was observed at *m*/*z* 277.1910 (C_16_H_24_N_2_O_2_ [M + H]^+^) and the fragmentation at 16.9 eV collision-induced dissociation (CID) energy resulted in methylmorpholine moiety detachment, which corresponded to the most abundant ion in the spectrum (*m*/*z* 100.0761, C_5_H_10_NO [M + H]^+^) and its further decomposition finally led to 2-(methylamino)prop-2-en-1-ylium ion formation (*m*/*z* 70.0658, C_4_H_8_N [M + H]^+^). In addition, parent molecule fragmentation also resulted in {3-[(2-hydroxyethyl)amino]propylidyne}oxidanium ion formation (*m*/*z* 116.0708, C_5_H_10_NO_2_ [M + H]^+^). The ion with *m*/*z* 190.1206 (C_12_H_16_NO [M + H]^+^) is another product of the morpholine moiety loss and its decomposition gradually led to the formation of the 2-methyl-1H-pyrrol-1-ium ion (*m*/*z* 82.0657, C_5_H_8_N [M + H]^+^).

The main metabolite, M1 (*m*/*z* 293.1860, C_16_H_25_N_2_O_3_ [M + H]^+^), was identified as a aliphatic hydroxyl derivative of molindone, 3-ethyl-2-(hydroxymethyl)-5-[(morpholin-4-yl)methyl]-1,5,6,7-tetrahydro-4H-indol-4-one (Figure S2). The hydroxylation occurred in aliphatic methyl side chain of the compound. The ion with *m*/*z* 275.1726 (C_16_H_23_N_2_O_2_ [M + H]^+^) testifies to the hydroxyl group detachment and its morpholine ring decomposition resulted in ions with *m*/*z* 221.1169 (C_12_H_17_N_2_O_2_ [M + H]^+^) formation and then in structural rearrangement of the 4,5-dihydro-1H-pyrano[3,4-b]pyrrolo[2,3-d]pyrrol-7-ylium radical ion (*m*/*z* 160.0605, C_9_H_8_N_2_O [M + H]^+^). The ion with *m*/*z* 100.0761, (C_5_H_10_NO [M + H]^+^) was the most visible peak, similar to that in the parent molecule spectrum.

^1^H NMR analysis also confirmed the introduction of the hydroxyl group in the methyl substituent in position 2 of the tetrahydroindol-4-one ring. First of all, absence of the distinct singlet signal of the above methyl group in the region δ 2.1 was observed, and simultaneously, the characteristic singlet for the hydroxymethyl group at δ 4.57 was registered. The presence of the ethyl group was confirmed by typical signals at δ 1.03 (t, *J* = 10 Hz) and δ 2.62 (q, *J* = 10 Hz). Two multiplets corresponding to the prochiral CH_2_, β to the pyrrole ring, appeared at δ 1.95/2.19. The observed multiplet at δ 3.82 (4H) belongs to the morpholine moiety while the remaining hydrogens appear as multiplets at δ 2.75–3.15.

The M2 metabolite (*m*/*z* 251.1762, C_14_H_23_N_2_O_2_ [M + H]^+^) was identified as a result of morpholine ring cleavage (Figure S3). The ion with *m*/*z* 178.1220 (C_11_H_16_NO [M + H]^+^) was the most abundant ion in the spectrum and testifies to morpholine ring loss. Its further fragmentation led to the formation of ions with *m*/*z* 160.1132 (C_11_H_14_NO [M + H]^+^) and *m*/*z* 122.0929 (C_8_H_12_N [M + H]^+^). The ion with *m*/*z* 74.0610 (C_3_H_8_NO [M + H]^+^) as the result of the aforementioned morpholine ring cleavage was also clearly visible.

The M3 metabolite (*m*/*z* 293.2012, C_16_H_25_N_2_O_3_ [M + H]^+^) was identified as another hydroxyl derivative of molindone with substitution most likely located in position C-7 in the cyclohexyl ring (Figure S4). The fragmentation lead to methylmorpholine moiety detachment (*m*/*z* 100.0760, C_5_H_10_NO [M + H]^+^) with its following degradation to ions with *m*/*z* 74.0586 (C_3_H_8_NO [M + H]^+^). The further decomposition of the ion with *m*/*z* 206.1131 (C_12_H_16_NO_2_), formed as a second fragment of morpholine moiety detachment from the parent compound, led to the formation of the ion with *m*/*z* 194.1113 (C_11_H_16_NO_2_ [M + H]^+^) with the remaining hydroxyl group.

The M4 metabolite (*m*/*z* 307.1654, C_13_H_23_N_2_O_4_ [M + H]^+^) was identified as a dihydroxy derivative of molindone (Figure S5). Dehydrogenation with a double bond formation in the tetrahydroindol-4-one ring is the second reaction that took place regarding the parent compound structure. Its fragmentation gradually led to both the removal of the hydroxyl groups, and the ion with *m*/*z* 289.1458 (C_16_H_21_N_2_O_3_ [M + H]^+^) testifies to the first step of this process. The ion with *m*/*z* 188.1040 (C_12_H_14_NO [M + H]^+^) was a product of the second hydroxyl group and morpholine moiety detachment simultaneously with the double bond retained. Furthermore, the ion with *m*/*z* 100.0759 (C_5_H_10_NO [M + H]^+^) was the most abundant ion in recorded spectrum.

The M5 metabolite (*m*/*z* 291.1694, C_16_H_23_N_2_O_3_ [M + H]^+^) was identified as a hydroxyl substituted molindone most likely located in position C-7 in the cyclohexyl ring (Figure S6). Dehydrogenation in the tetrahydroindol-4-one ring was the second reaction that occurred with regard to the parent compound. The most abundant ion with *m*/*z* 100.0760 (C_5_H_10_NO [M + H]^+^) belonged to the detached methylmorpholine moiety, whereas the fragment with *m*/*z* 204.1039 (C_12_H_14_NO_2_ [M + H]^+^) testifies to the retention of the double bond and hydroxyl group in the second part of the fragmented metabolite structure. Further fragmentation of this ion consisted of two paths. One led to hydroxyl group detachment and the ion with *m*/*z* 176.1069 (C_11_H_14_NO[M + H]^+^) was formed in this case. The second path assumed rearrangement with the formation of the ion at *m*/*z* 162.0896 (C_10_H_12_NO [M + H]^+^), followed by additional rearrangement resulting in the formation of the 5-ethyl-6-(hydroxymethyl)pyridin-3-ylium ion (*m*/*z* 136.0735, C_8_H_10_NO [M + H]^+^)

The protonated molecular ion for the M6 metabolite was observed at *m*/*z* (275.1754, C_16_H_23_N_2_O_2_ [M + H]^+^). Measured *m*/*z* and the generated formula indicated the presence of an unsaturated bond located in the tetrahydroindol-4-one ring. The mild fragmentation at 10.0 eV CID energy resulted in methylmorpholine moiety detachment, which corresponded to the ion with *m*/*z* 100.0769 (C_5_H_10_NO [M + H]^+^). The rest of the metabolite structure with *m*/*z* 188.1036 (C_12_H_14_NO) was also visible (Figure S7).

### 2.4. Transformation Pathways

**Figure 4:**
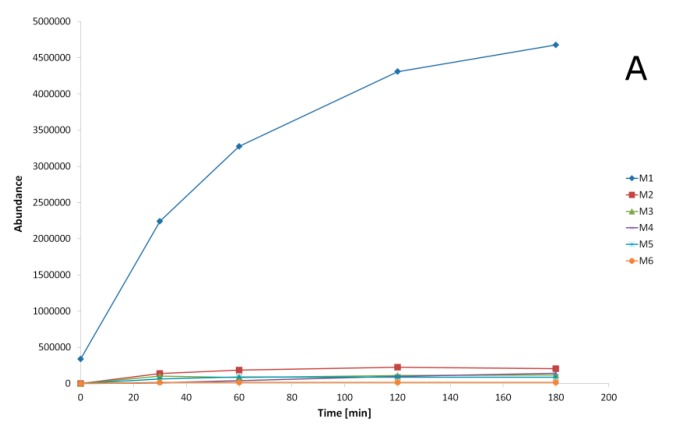

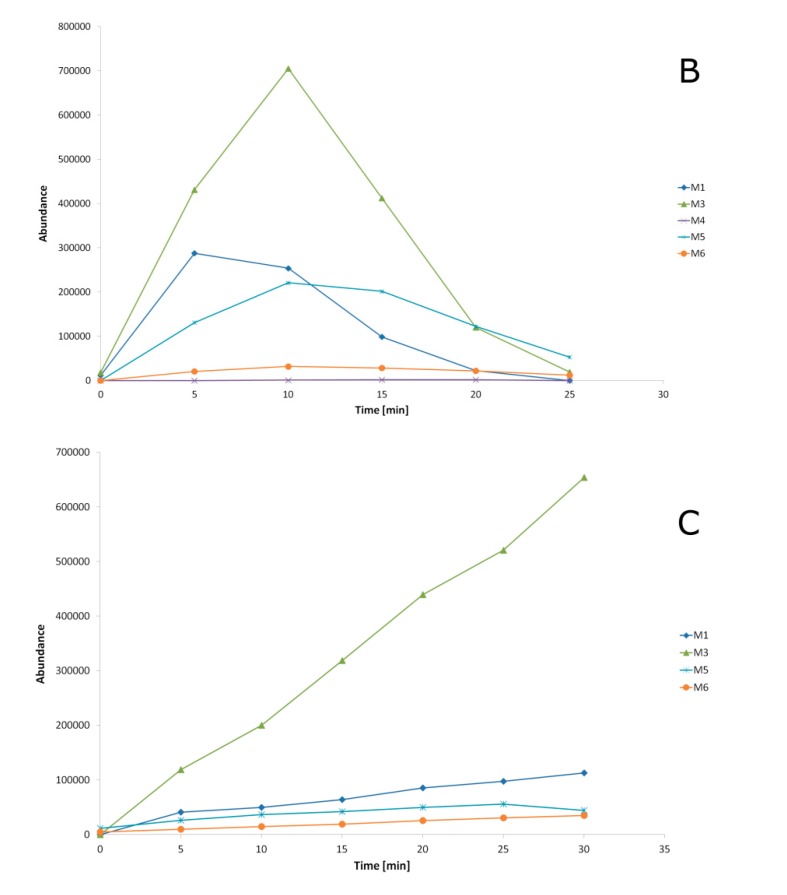
Evolution profiles of molindone metabolites formed during HLM incubation (**A**), WO_3_ assisted (**B**), and WS_2_ assisted photochemical reaction (**C**).

**Figure 5:**
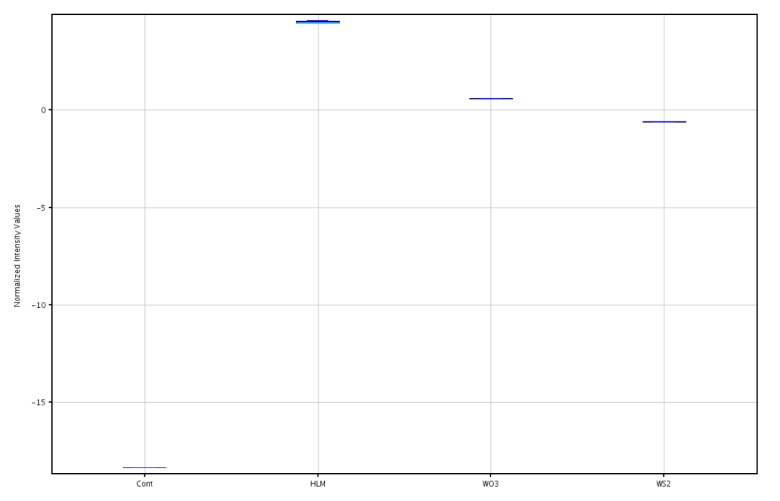
Box whisker plot of the relative intensity values of the main metabolite (M1) in the analyzed profiles (Control group, HLM, WO_3_, WS_2_) of molindone.

The study resulted in the confirmation of one major and five minor metabolites of molindone obtained during microsomal incubation. As shown in Table 1, the parent compound underwent several metabolic reactions: hydroxylation (M1 and M3), dealkylation (M2), combination of hydroxylation and dehydrogenation (M4 and M5), and dehydrogenation (M6). It was noticeable that the aliphatic hydroxylation process was found to be the process responsible for the main metabolite (M1) formation (Figure 4A), and this reaction was successfully simulated with the use of both photocatalysts. The multivariate chemometric comparison of the distribution of this metabolite in the analyzed photocatalytic samples also confirmed their similarity to the biological experiment (Figure 5). Moreover, the WO_3_ assisted photocatalysis allowed us to observe four minor metabolites (M3, M4, M5, and M6), whereas the WS_2_ assisted photocatalysis allowed to observe three minor metabolites (M3, M5, and M6). It should be also noted that the photocatalytic methods significantly improved the identification of the minor metabolites due to their higher abundances and better detection with the use of the MS method.

**Figure 6:**
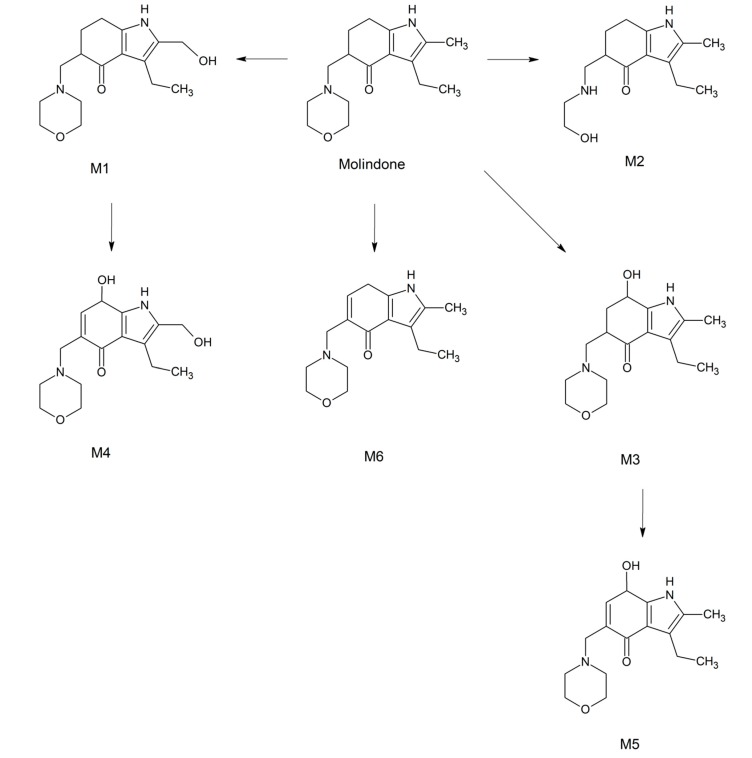
The proposed metabolic pathway of molindone.

The proposed metabolic pathway of molindone is presented in Figure 6.

## 3. Materials and Methods

### 3.1. Chemicals and Reagents

Molindone hydrochloride was obtained from Toronto Chemicals (Toronto, ON, Canada). Water (LC-MS Ultra grade), β-nicotinamide adenine dinucleotide 2′-phosphate reduced tetrasodium salt hydrate (NADPH), human liver microsomes (HLMs), sodium phosphate monobasic monohydrate salt, sodium phosphate dibasic anhydrous salt, and tungsten (VI) oxide nanopowder < 100 nm particle size were obtained from Sigma-Aldrich (St. Louis, MO, USA). Tungsten disulfide (IV) nanopowder 40–80 nm particle size, amorphous was obtained from U.S. Research Nanomaterials, Inc. (Houston, TX, USA). Acetonitrile (hypergrade for LC-MS), were purchased from Merck (Darmstadt, Germany) and 98% formic acid (mass spectroscopy grade) was obtained from Fluka (Taufkirchen, Germany).

### 3.2. Photocatalytic Experiments

The photocatalytic reactions were performed in the aqueous solution at a concentration of 25 µM of the tested drugs. The applied catalyst loadings were set to 100 mg L^−1^ of WO_3_ and 100 mg L^−1^ of WS_2_. For all experiments, suspensions were transferred into 3.5 mL quartz caped cells (l = 1 cm) and stirred at 500 rpm (microstirrer Cimarel: Telemodul, Thermo Electron LED GmbH, Osterode am Harz, Germany) in the dark for 60 min to achieve adsorption–desorption equilibrium. Next, reaction cells were mounted horizontally in an Atlas Suntest CPS+ photostability chamber with a D65 filter (Linsengericht, Hesse, Germany), and irradiated simultaneously with stirring. The irradiance was set to 765 W m^−2^, which corresponded to an energy dose of 2854 kJ m^−2^ h^−1^. The temperature in the chamber was controlled and kept below 35 °C. Aliquots (100 µL) were collected at proper intervals and the following time ranges were set to: 0–25 min for WO_3_ and 0–30 min for WS_2_ photocatalytic experiments. All suspensions were then centrifuged at 15,000 rpm for 5 min (Eppendorf 5424R, Germany), 50 μL aliquots were collected, and UHPLC-ESI-Q-TOF analysis was performed.

In order to perform the isolation of the main metabolite (M1), 2 mg mL^−1^ solution of molindone with the addition of WO_3_ was irradiated for 6 h under the same conditions and next semi-preparative chromatography was realized.

### 3.3. In Vitro Simulation of Metabolism by Human Liver Microsomes

Phase I metabolism reactions were performed in vitro with the use of the HLM fraction. The incubation system consisted of 50 μM substrate, 55 mM phosphate buffer (pH 7.4), and 0.5 mg mL^−1^ microsomes. The incubation system was pre-incubated at 37 °C for 2 min (Eppendorf ThermoMixer C equipped with Eppendorf ThermoTop, Hamburg, Germany) and then the metabolic reactions were initiated by the addition of 10 μL NADPH (20 mM). Total volume of the reaction suspension was 200 μL and no organic solvent was added into the system. The reaction was terminated after 0, 30, 60, 120, and 180 min of incubation with 200 μL of ice-cold acetonitrile:methanol mixture (1:1). Next, the precipitated samples were centrifuged at 15,000 rpm for 10 min at 4 °C (Eppendorf 5424R, Hamburg, Germany) and the supernatants (50 μL) were transferred into autosampler vials for LC-MS analysis. The negative control samples were prepared as described above without the addition of NADPH solution.

### 3.4. Analytical Procedures

The LC-MS analysis was performed with the use of an Agilent Accurate-Mass Q-TOF LC/MS G6520B system with a dual electrospray (DESI) ionization source and Infinity 1290 ultra-high-pressure liquid chromatography system consisting of binary pump G4220A, FC/ALS thermostat G1330B, autosampler G4226A, DAD detector G4212A, TCC G1316C module (Agilent Technologies, Santa Clara, CA, USA), and a Kinetex C18 (2.1 × 50 mm, dp = 1.7 μm) column with a C18 precolumn guard (Phenomenex, Torrance, CA, USA). A mixture of water containing a 0.1% solution of formic acid with 5% addition of acetonitrile (A), and acetonitrile (B) was used as a mobile phase. The gradient elution was carried out at a constant flow 0.3 mL min^−1^ from 95%A (5%B) to 85%A (15%B) for 0–9 min, then to 5%A (95%B) 9–9.50 min, and a 1.50 min post time was performed to return to initial conditions. The injection volume was 2.0 µL and the column temperature was maintained at 35 °C. MassHunter workstation software in version B.08.00 was used for the control of the system, data acquisition, qualitative, and quantitative analysis.

The optimization of the instrument conditions started with the proper tuning of the Q-TOF detector in a positive mode with the use of an Agilent ESI-L tuning mix in the extended dynamic range (2 GHz). The following instrument settings were applied: gas temp.: 325 °C, drying gas: 10 L/min, nebulizer pressure: 40 psig, capillary voltage: 3500 V, fragmentor voltage: 175 V, skimmer voltage: 65 V, octopole 1 RF voltage: 750 V.

Data acquisition was performed in centroids with the use of TOF (MS) and auto MS/MS mode. The spectral parameters for both modes were: mass range: 60–950 m/z and the acquisition rate: 2.0 spectra/s. To ensure accuracy in the mass measurements, a reference mass correction was used and the masses of 121.050873 and 922.009798 were used as lock masses.

Semi-preparative chromatography was executed with the use of a Shimadzu UFLC (ultra-fast liquid chromatography) system consisting of: pump LC-20 AD, degassing DGU-20A5R, column oven CTO-10AS VP, DAD detector SPD-M20A (Shimadzu Corporation, Kyoto, Japan), and Zorbax SB-C18 (9.4 × 150 mm, dp = 5 μm) semi-preparative column (Agilent, Santa Clara, CA, USA). A mixture of water with the addition of 0.1% solution of formic acid and acetonitrile (96.5:3.5, *v*/*v*) was used as the mobile phase. The isocratic elution was carried out at a flow of 4 mL min^−1^ for 65 min. The injection volume was 500 μL, and the column temperature was maintained at 30 °C. A Gilson FC 203B (Gilson Inc., Middleton, WI, USA) fraction collector was used to collect the proper fraction containing the M1 metabolite. The received fifteen fractions were pooled and evaporated using a Hei-VAP Value G3 rotary evaporator (Heidolph, Schwabach, Germany) under 50 mbar vacuum at a temperature of 35 °C. The residue was dissolved in 2 mL of acetonitrile and evaporated under the nitrogen stream. Identity and purity of the residuals were controlled using the LC-MS, and then NMR analyses were performed.

The NMR spectra were recorded on a Bruker Avance III (500 MHz, Bruker, Billerica, MA, USA) spectrometer. Chemical shifts are given in parts per million (δ) downfield from tetramethylsilane as the internal standard. Methanol-*d*_4_ was used as the solvent.

### 3.5. Chemometric Analysis

Four metabolism experiments: HLM (after 120 min of incubation), control sample (HLM without NADPH), WO_3_ photocatalytic (after 5 min of irradiation), and WS_2_ photocatalytic (after 30 min of irradiation) were performed in six replications for each experiment. In this manner, a set of twenty four samples for four different experiments for one of the tested drugs was obtained. For all of these samples, high resolution LC-MS analysis was performed in TOF (MS) mode and their specific chromatographic/spectral profiles were recorded. The molecular feature extraction (MFE) algorithm from the Mass Hunter Qualitative Analysis software version B.06.00 (Agilent) was used for data background ion noise cleaning and to extract the list of the ion characteristics for the metabolite profiles of the analyzed substances. The MFE parameters were optimized and the following settings were applied: single charge state of the analyzed ions, more than 2000 counts for the compound filter; isotope model: common organic molecules with peak spacing tolerance 0.0025 *m*/*z*. In order to perform the multivariate chemometric analysis, the obtained results were then exported to the Mass Profiler Professional (MPP) software version 12.61 (Agilent and Strand Life Sciences Pvt. Ltd., Santa Clara, CA, USA. With the use of this software, the data were normalized and aligned, and the principal component analysis (PCA) was performed.

## 4. Conclusions

The characterization of six, previously not described metabolites of molindone was the main accomplishment of this study. Tungsten disulfide was successfully introduced in the drug metabolism simulation, however, tungsten oxide (VI) provided a better reproduction of the metabolic profile in accordance with applied multivariate chemometric comparison. Moreover, the photocatalysis was utilized for the first time as a noteworthy technique in drug metabolite synthesis in order for possible future isolation and research.

The obtained high-resolution MS/MS spectra were found to hold a peculiar value in drug metabolite identification. The UHPLC-ESI-Q-TOF MS combined system proved its usefulness as a preferred analytical tool in drug metabolism studies.

## Figures and Tables

**Table 1:** Q-TOF accurate mass elemental composition and MS/MS fragmentation and of the analyzed substances.

Name	Reaction Type	Retention Time [min]	Measured Mass [*m*/*z*]	Theoretical Mass [*m*/*z*]	Mass Error [ppm]	Molecular Formula [M + H]^+^	MS/MS Fragmentation [*m*/*z*]	Fragmentation Ion Formula [M + H]^+^
MO	–	6.53	277.1910	277.1910	0.00	C_16_H_24_N_2_O_2_	190.1206 176.1057 116.0708 100.0761 82.0657 70.0658	C_12_H_16_NO C_11_H_14_NO C_5_H_10_NO_2_ C_5_H_10_NO C_5_H_8_N C_4_H_8_N
M1	Al-OH	2.50	293.1860	293.1859	0.34	C_16_H_25_N_2_O_3_	275.1726 221.1169 160.0605 100.0761 70.0660	C_16_H_23_N_2_O_2_ C_12_H_17_N_2_O_2_ C_9_H_8_N_2_O C_5_H_10_NO C_4_H_8_N
M2	Dealk	5.24	251.1762	251.1754	3.18	C_14_H_23_N_2_O_2_	178.1220 160.1132 122.0929 74.0610	C_11_H_16_NO C_11_H_14_NO C_8_H_12_N C_3_H_8_NO
M3	Al-OH	4.90	293.2012	293.1859	52.18	C_16_H_25_N_2_O_3_	206.1131 194.1113 188.1022 100.0760 74.0586	C_12_H_16_NO_2_ C_11_H_16_NO_2_ C_12_H_14_NO C_5_H_10_NO C_3_H_8_NO
M4	Al-2OH Dehydrog	3.32	307.1654	307.1652	0.65	C_13_H_23_N_2_O_4_	289.1458 188.1040 100.0759 84.0793	C_16_H_21_N_2_O_3_ C_12_H_14_NO C_5_H_10_NO C_5_H_10_N
M5	Al-OH Dehydrog	3.70	291.1694	291.1703	−3.09	C_16_H_23_N_2_O_3_	204.1039 176.1069 162.0896 136.0735 100.0760	C_12_H_14_NO_2_ C_11_H_14_NO C_10_H_12_NO C_8_H_10_NO C_5_H_10_NO
M6	Dehydrog	4.30	275.1754	275.1754	0.00	C_16_H_23_N_2_O_2_	188.1036 100.0769	C_12_H_14_NO C_5_H_10_NO

Abbreviations: Al-OH—aliphatic hydroxylation, Al-2OH—aliphatic dihydroxylation, Dealk—dealkylation, Dehydrog—dehydrogenation.

## Supplementary Materials

The following are available online: Figure S1: MS/MS spectrum and fragmentation pathway of molindone, Figure S2: MS/MS spectrum and fragmentation pathway of M1, Figure S3: MS/MS spectrum and fragmentation pathway of M2, Figure S4: MS/MS spectrum and fragmentation pathway of M3, Figure S5: MS/MS spectrum and fragmentation pathway of M4, Figure S6: MS/MS spectrum and fragmentation pathway of M5, Figure S7: MS/MS spectrum and fragmentation pathway of M6.
