# Supplementary material for: Mimicking of Phase I Metabolism Reactions of Molindone by HLM and Photocatalytic Methods with the Use of UHPLC-MS/MS
Source: Molecules. 2020 Mar 17;25(6):1367. doi: 10.3390/molecules25061367 (PMC7144366; doi:10.3390/molecules25061367)
Supplement: Supplementary file 1 [file molecules-25-01367-s001.pdf]

Supplementary material

Photocatalysis as a new approach to profiling, synthesis and isolation of drugs metabolites: an example of molindone

Maciej Gawlik[1], Vladimir Savic[2], Milos Jovanovic[2], Robert Skibiński[1*]

[1]Department of Medicinal Chemistry, Faculty of Pharmacy, Medical University of Lublin, Jaczewskiego 4, 20-090 Lublin, Poland

[2]Department of Organic Chemistry, Faculty of Pharmacy, University of Belgrade, Vojvode Stepe 450, Belgrade, Serbia

[*] Corresponding author. Tel.: +48 81 4487390; fax: +48 81 4487380.

E-mail address: robert.skibinski@umlub.pl

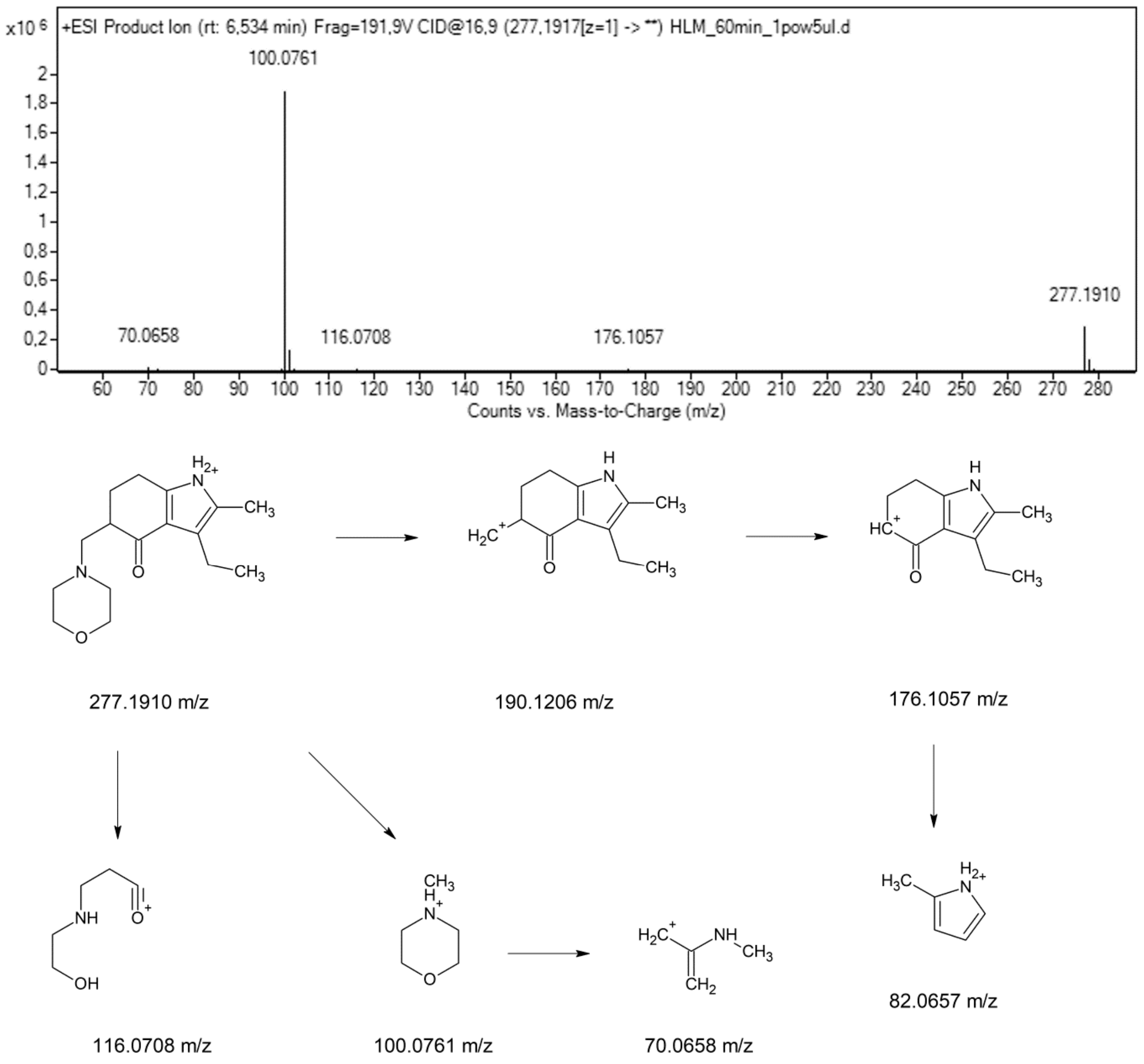

Fig. S1. MS/MS spectrum and fragmentation pathway of molindone

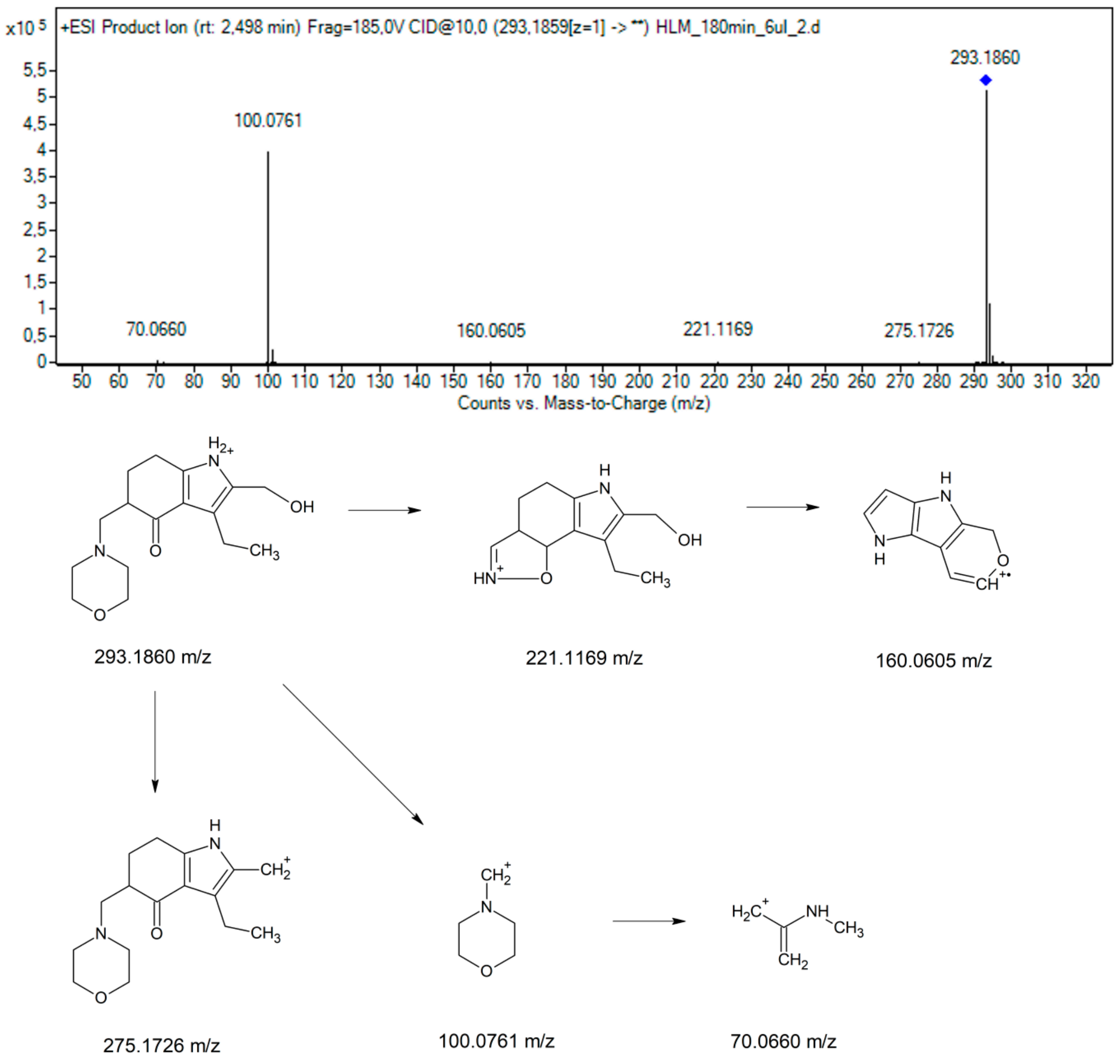

Fig. S2. MS/MS spectrum and fragmentation pathway of M1

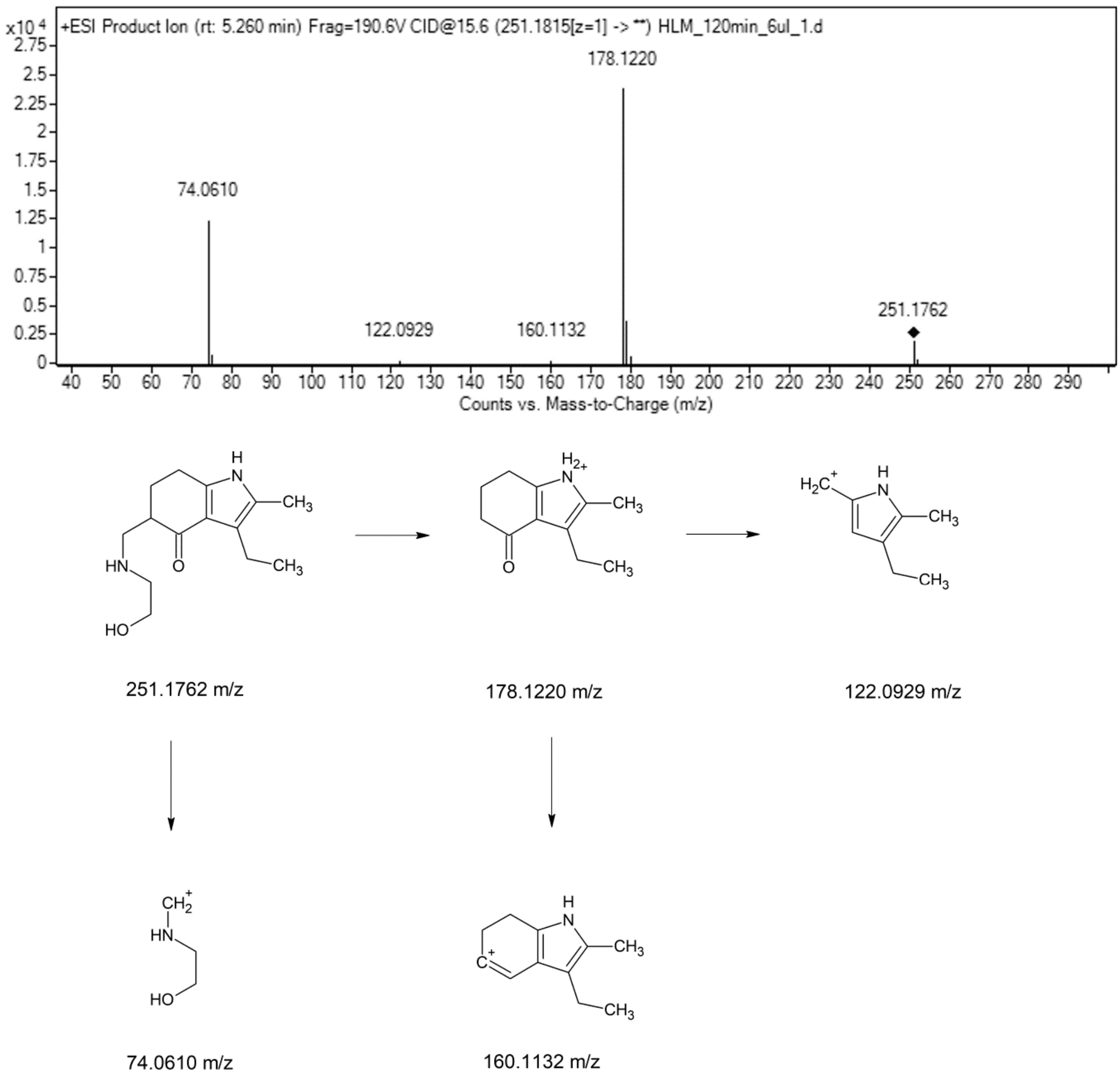

Fig. S3. MS/MS spectrum and fragmentation pathway of M2

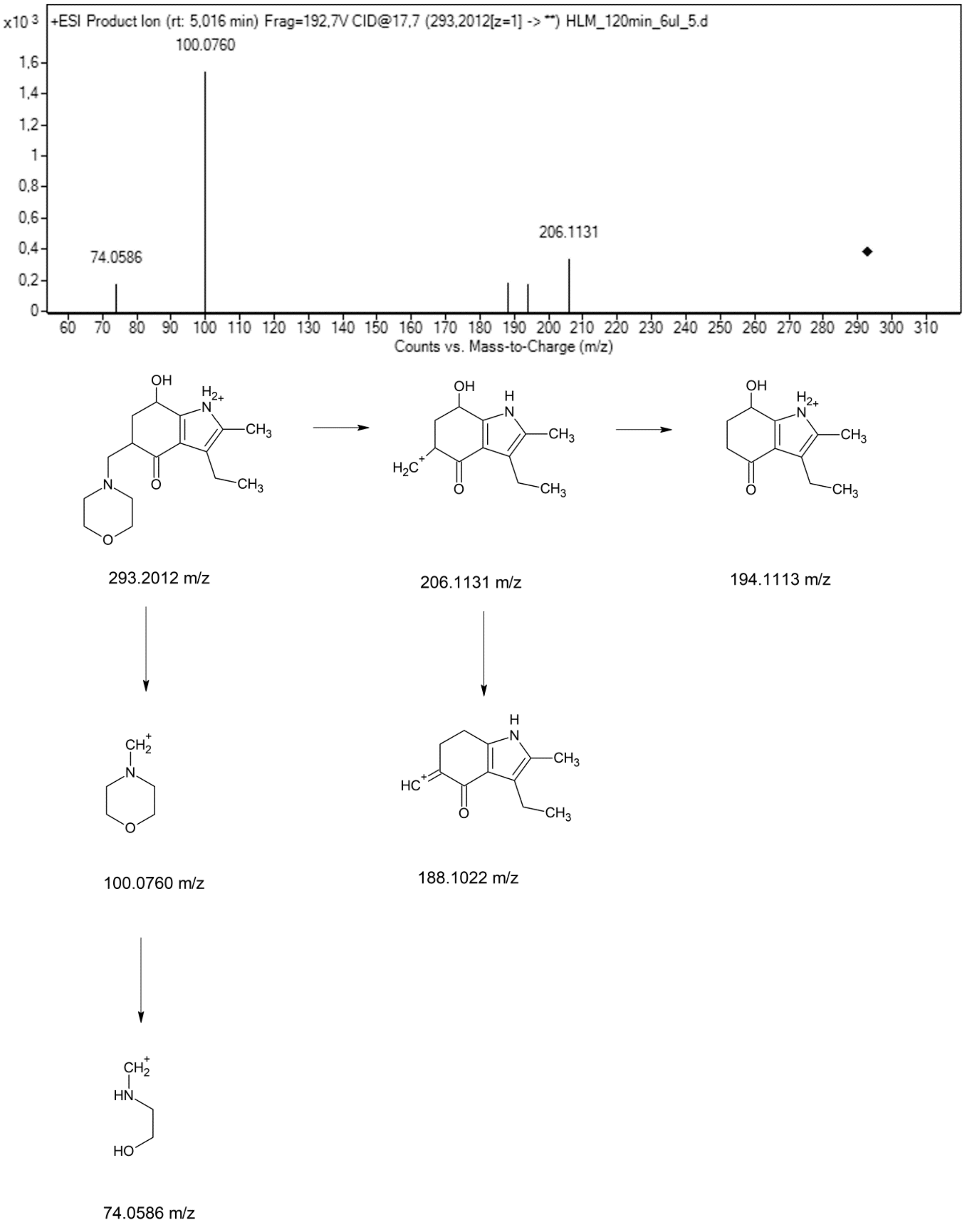

Fig. S4. MS/MS spectrum and fragmentation pathway of M3

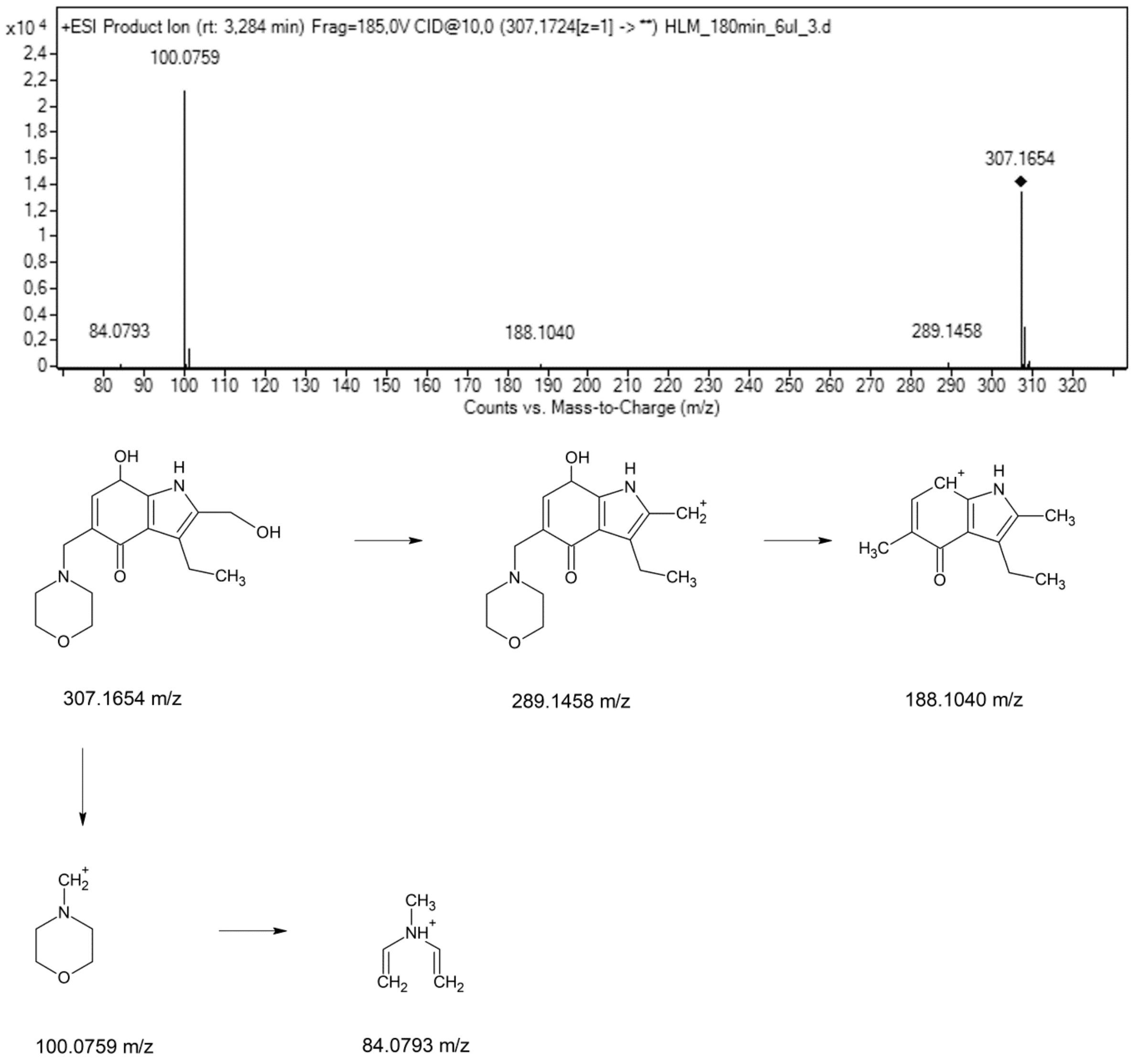

Fig. S5. MS/MS spectrum and fragmentation pathway of M4

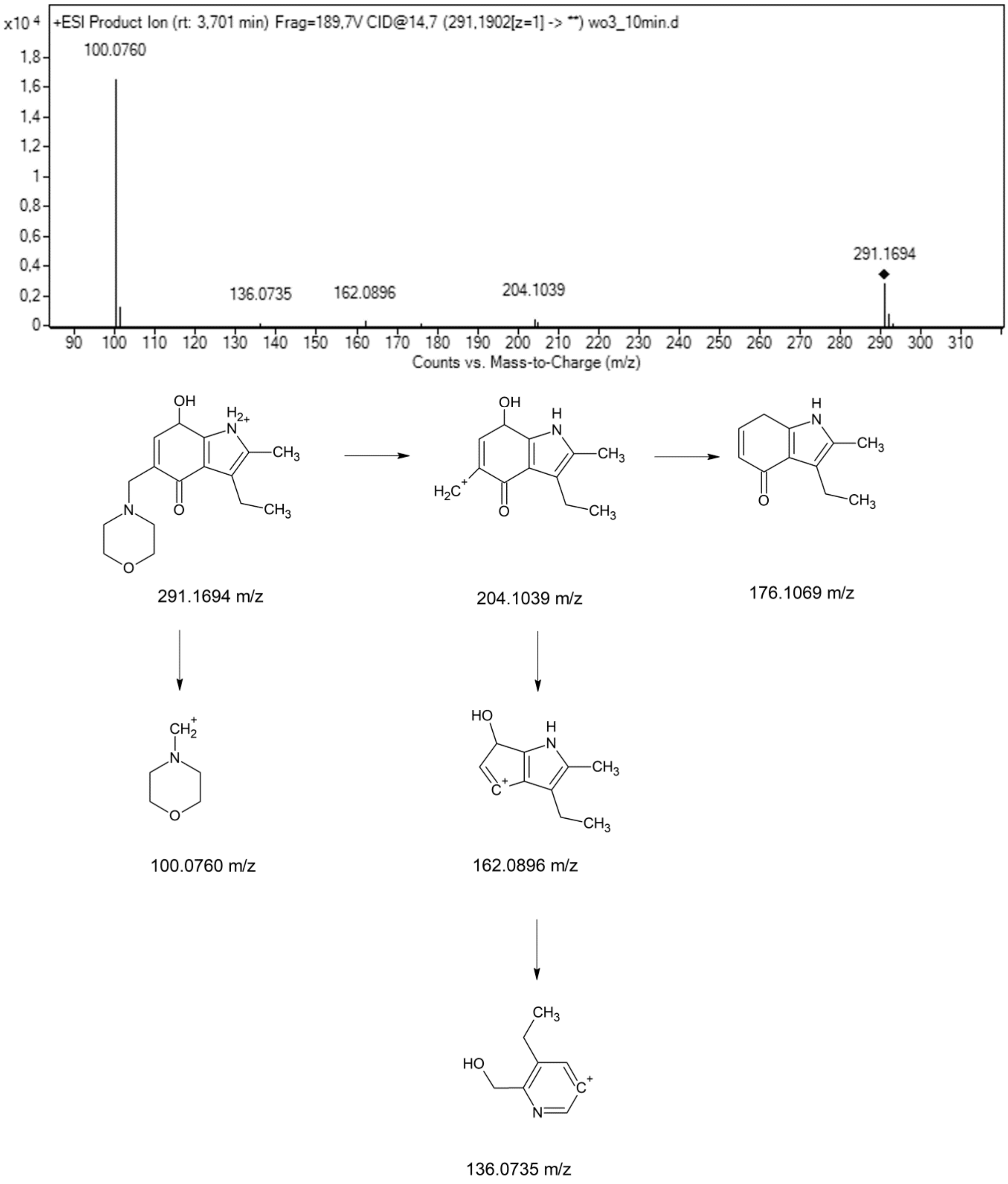

Fig. S6. MS/MS spectrum and fragmentation pathway of M5

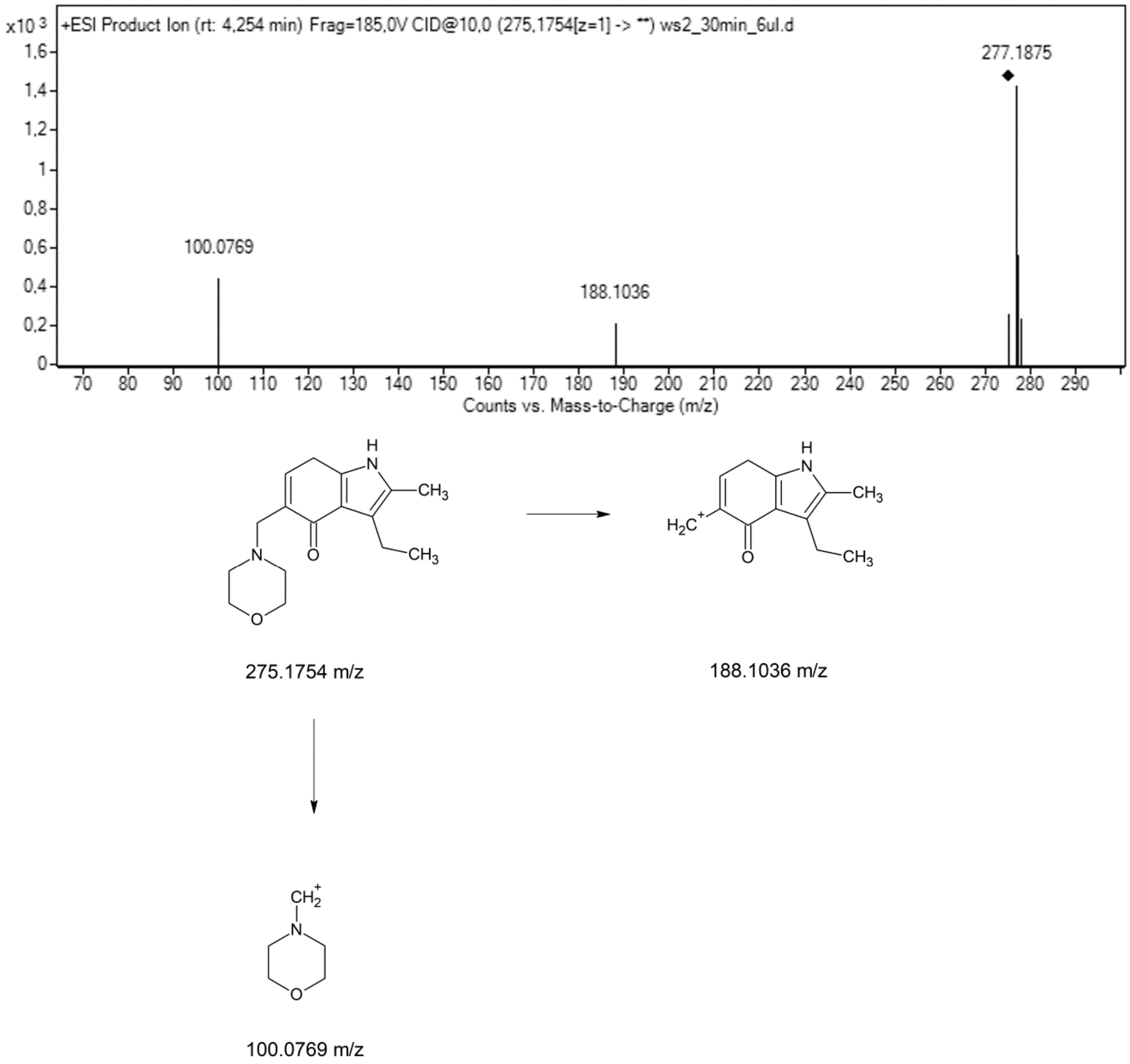

Fig. S7. MS/MS spectrum and fragmentation pathway of M6
